# Resveratrol Induces Myotube Development by Altering Circadian Metabolism via the SIRT1-AMPK-PP2A Axis

**DOI:** 10.3390/cells13121069

**Published:** 2024-06-20

**Authors:** Natalie Avital-Cohen, Nava Chapnik, Oren Froy

**Affiliations:** Institute of Biochemistry, Food Science and Nutrition, Robert H. Smith Faculty of Agriculture, Food and Environment, The Hebrew University of Jerusalem, Rehovot 76100, Israel; natalie.avital@mail.huji.ac.il (N.A.-C.); nava.chapnik@mail.huji.ac.il (N.C.)

**Keywords:** resveratrol, mTOR, clock, circadian, metabolism, muscle, PP2A

## Abstract

Resveratrol is a polyphenol known to have metabolic as well as circadian effects. However, there is little information regarding the metabolic and circadian effect of resveratrol on muscle cells. We sought to investigate the metabolic impact of resveratrol throughout the circadian cycle to clarify the associated signaling pathways. C2C12 myotubes were incubated with resveratrol in the presence of increasing concentrations of glucose, and metabolic and clock proteins were measured for 24 h. Resveratrol led to SIRT1, AMPK and PP2A activation. Myotubes treated with increasing glucose concentrations showed higher activation of the mTOR signaling pathway. However, resveratrol did not activate the mTOR signaling pathway, except for P70S6K and S6. In accordance with the reduced mTOR activity, resveratrol led to advanced circadian rhythms and reduced levels of pBMAL1 and CRY1. Resveratrol increased myogenin expression and advanced its rhythms. In conclusion, resveratrol activates the SIRT1-AMPK-PP2A axis, advances circadian rhythms and induces muscle development.

## 1. Introduction

The mammalian circadian clock is located in the suprachiasmatic nuclei (SCN) of the anterior hypothalamus. Light perceived by the retina synchronizes SCN neurons that relay the information to peripheral tissues via neural and humoral signals [1]. The core clock consists of CLOCK (circadian locomotor output cycles protein kaput) and BMAL1 (brain and muscle Arnt-like protein-1) that activate transcription of many tissue-specific genes by binding to enhancer elements. PERIODs (PERs) and CRYPTOCHROMEs (CRYs) form the negative feedback loop that inhibits CLOCK:BMAL1-mediated transcription [2]. The synchronized expression of circadian clock genes orchestrates the expression of hormones, enzymes, nuclear receptors and transporters involved in metabolism [3]. Disturbances in circadian rhythms have been shown to disrupt metabolism [4,5].

Resveratrol is a polyphenolic compound found in grape skin, peanuts and berries [6,7]. It has been shown to have diverse benefits including anti-inflammatory, anti-oxidant, anti-diabetic and anti-carcinogenic powers [6,7]. Circadian rhythms have been shown to be affected by resveratrol [8,9]. This is achieved by activating Sirtuin 1 (SIRT1) [10,11], an important metabolic factor that alters circadian rhythms by deacetylating histones and activating the CLOCK:BMAL1-mediated transcription [12].

SIRT1 activation is usually accompanied by AMP-activated protein kinase (AMPK) activation [13]. This activation leads to the inhibition of the mTOR signaling pathway [14]. Indeed, some studies report that resveratrol leads in addition to SIRT1 activation and also to AMPK activation [15]. In addition, resveratrol has been shown to activate protein phosphatase 2A (PP2A) [8,16]. However, PP2A has been reported to dephosphorylate AMPK, leading to inactivation of the latter [17,18]. As the activation of AMPK, SIRT1 and PP2A are influenced by the cellular metabolic state, we studied the effect of resveratrol in the presence of low, normal and high glucose concentrations. These analyses were performed for 24 h to elucidate the signaling pathways involved in myotube development.

## 2. Materials and Methods

### 2.1. Cell Culture and Treatments

C2C12 myoblasts were grown in Dulbecco’s Modified Eagle’s Medium (DMEM) (Biological Industries, Beit Haemek, Israel) supplemented with 10% fetal bovine serum, 1% antibiotics solution (Biological Industries) and 5% CO_2_ at 37 °C. When cells were confluent, the medium was replaced with DMEM supplemented with 2% horse serum every day for a period of 48–72 h until differentiation was achieved. Afterward, the cells were synchronized with 1 mM dexamethasone (Sigma, Rehovot, Israel) for 1 h, and then the medium was exchanged for fresh DMEM containing 50 μM resveratrol (Sigma) for 6 h based on previous studies [8]. For control, medium was replaced with fresh DMEM supplemented with 2.75 mM, 5.5 mM or 25 mM glucose. To measure average daily levels, cells were harvested in triplicates every 6 h for 24 h. We performed three independent experiments.

### 2.2. Cell Viability Assay

To evaluate cell viability in relation to redox potential, we utilized 3-(4,5-dimethylthiazol-2-yl)-2,5-diphenyltetrazolium bromide (MTT). After a 24 h incubation with 50 µM resveratrol, the medium was removed, and the cells were treated with 0.5 mg/mL MTT in DMEM for 1 h at 37 °C. The MTT solution was then discarded, followed by the addition of 1 mg/mL dimethylsulfoxide. Optical density was measured at 595 nm using a microplate reader.

### 2.3. Western Blot Analyses

Cells were homogenized in 200 μL lysis buffer (pH 7.8, 5% glycerol, 1% Triton X-100, 20 mM Tris, 50 nM phenylmethylsulfonyl fluoride (PMSF), 145 mM NaCl, 50 μM sodium fluoride (NaF) and protease inhibitor (Sigma)). Samples were subjected to 10% SDS-polyacrylamide gel electrophoresis, after which proteins were semi-dry transferred onto nitrocellulose membranes. The blots were then incubated with antibodies against AMP-activated protein kinase (AMPK) and its phosphorylated form (pAMPK), protein phosphatase 2A (PP2A), pPP2A, SIRT1, acetyl CoA carboxylase (ACC), pACC, BMAL1, pBMAL1, AKT, P70S6K, pP70S6K, S6 and pS6 (Cell Signaling Technology, Beverly, MA, USA), ACTIN, pAKT, mTOR, pmTOR, MYOGENIN, CLOCK and CRY1 (Santa Cruz Biotechnologies, Santa Cruz, CA, USA). Following several washes, the membranes were treated with a horseradish peroxidase-conjugated secondary antibody (Pierce, Rockford, IL, USA). To detect actin, which served as the loading control, an anti-mouse antibody (MP Biomedicals, Solon, OH, USA) was used. Bands were quantified by scanning and densitometry and expressed as arbitrary units.

### 2.4. RNA Extraction and Quantitative Real-Time PCR

Total RNA was extracted using TRI Reagent (Sigma) and was DNase I-treated using RQ1 DNase (Promega, Madison, WI, USA). Reverse-transcription was performed using qScript cDNA synthesis kit (Quanta BioSciences, Gaithersburg, MD, USA) and random hexamers (Promega). Quantitative real-time PCR was conducted with primers (Supplementary Table S1) that spanned exon–exon boundaries, using the ABI Prism 7300 Sequence Detection System (Applied Biosystems, Foster City, CA, USA). Gene expression levels were normalized to actin. The reaction conditions were set as follows: 95 °C for 3 min, 95 °C for 10 s, and 60 °C for 45 s. The fold change in target gene expression was determined using the 2^−∆∆Ct^ relative quantification method (Applied Biosystems).

### 2.5. Statistical Analyses

All results are expressed as mean ± SE. For all analyses, the significance level was set at *p* < 0.05 using Student’s *t*-test or Tukey’s honestly significant difference (HSD). One-way ANOVA was performed to analyze the circadian pattern of genes and proteins with several time-points. Statistical analysis was performed with JMP software (version Pro 16) (SAS Institute Inc., Cary, NC, USA). Additional analyses of circadian rhythmicity were performed using Circwave software (version 1.4) (Circadian Rhythm Laboratory, University of Groningen, Groningen, The Netherlands).

## 3. Results

### 3.1. Effect of Resveratrol on the SIRT1-AMPK-PP2A Axis in Myotubes

**Figure 1:**
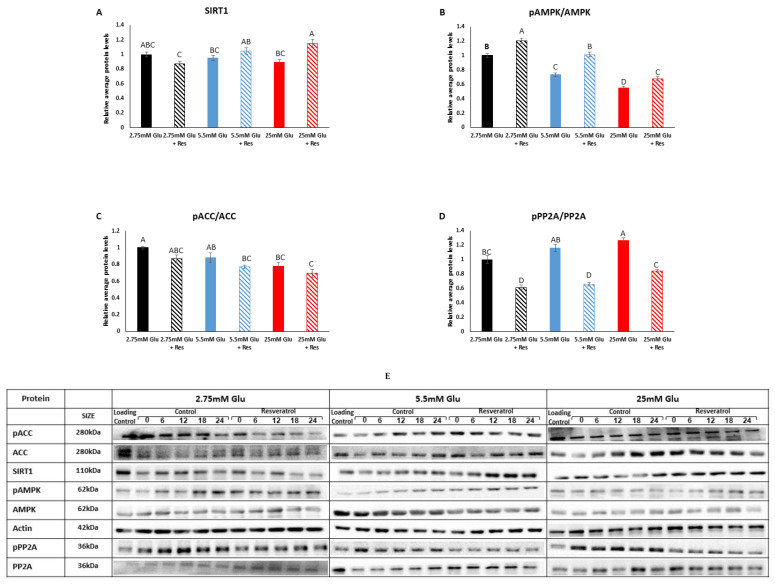
Effect of resveratrol on metabolic factors. (**A**) SIRT1. (**B**) pAMPK/AMPK. (**C**) pACC/ACC. (**D**) pPP2A/PP2A. (**E**) Representative Western blots of all proteins at different glucose concentrations around the circadian cycle. C2C12 myotubes were incubated with resveratrol for 6 h and analyzed for an additional 24 h. Western blots were performed to determine protein levels. All time-points are expressed as average daily levels. Within each panel, protein levels display statistical significance (*p* < 0.05) only when bars are presented with different letters.

We first measured the levels of SIRT1, AMPK and PP2A, known to be affected by resveratrol, under low, normal and high glucose concentrations. SIRT1 protein levels did not change under the different glucose concentrations (*p* > 0.05) (Figure 1A). However, resveratrol led to increased SIRT1 expression in the high-glucose group (*p* = 0.005). As SIRT1 is activated by AMPK and several reports have shown AMPK activation in the presence of resveratrol, we next measured AMPK activity. Indeed, the ratio of phosphorylated AMPK to total AMPK (pAMPK/AMPK) was high after resveratrol treatment (*p* < 0.0001 for 2.75 mM and 5.5 mM, *p* = 0.03 for 25 mM) (Figure 1B), indicating AMPK activation under these conditions. As expected, the pAMPK/AMPK ratio decreased under increasing glucose concentrations (*p* < 0.0003) (Figure 1B), as AMPK is activated under low cellular energy levels. The reduced activation of AMPK under increasing glucose concentrations was mirrored by the reduced ratio of phosphorylated acetyl CoA carboxylase (pACC) to ACC, the target of AMPK, between 2.75 mM and 25 mM glucose (*p* = 0.0045) (Figure 1C). Reduced pACC/ACC ratio indicates activation of ACC, the rate-limiting enzyme in fatty acid synthesis, which is expected under increasing glucose concentrations. Resveratrol treatment did not affect the pACC/ACC ratio (*p* > 0.05) (Figure 1C). In light of AMPK activation, we measured PP2A, whose active form dephosphorylates and, hence, inhibits AMPK. Resveratrol treatment led to lower levels of phosphorylated PP2A, leading to a more active PP2A (*p* < 0.0001) (Figure 1D). Interestingly, increasing glucose concentrations led to a high ratio of pPP2A/PP2A, indicating inhibition, similarly to the effect on AMPK. Thus, surprisingly, resveratrol led to both AMPK and PP2A activation. PP2A inhibition under increasing glucose concentrations matches the activation achieved with ACC, its target for dephosphorylation. Taken together, these results suggest that resveratrol leads to the activation of AMPK-SIRT1 as well as opposing phosphatase PP2A.

### 3.2. Effect of Resveratrol on the mTOR Signaling Pathway in Myotubes

**Figure 2:**
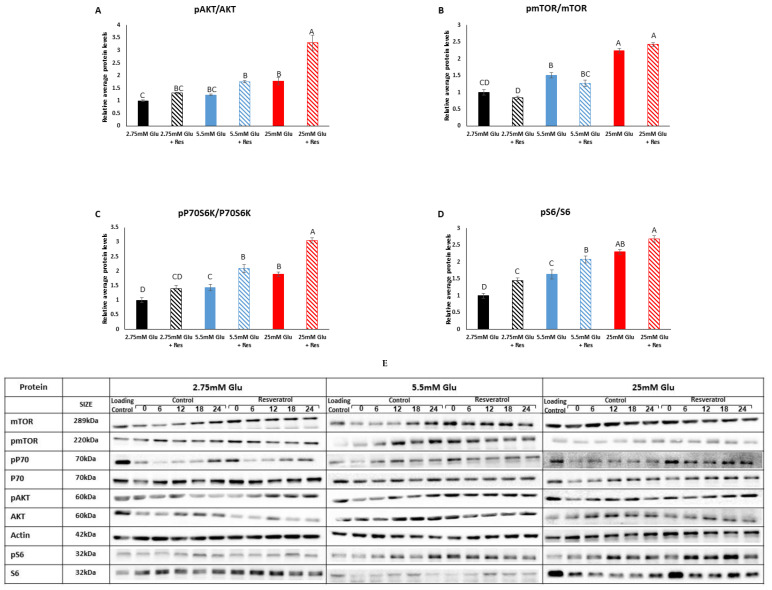
Effect of resveratrol on the mTOR signaling pathway. (**A**) pAKT/AKT. (**B**) pmTOR/mTOR. (**C**) pP70S6K/P70S6K. (**D**) pS6/S6. (**E**) Representative Western blots of all proteins at different glucose concentrations around the circadian cycle. C2C12 myotubes were incubated with resveratrol for 6 h and analyzed for an additional 24 h. Western blots were performed to determine protein levels. All time-points are expressed as average daily levels. Within each panel, protein levels display statistical significance (*p* < 0.05) only when bars are presented with different letters.

Myotubes treated with increasing glucose concentrations showed higher activation of the mTOR signaling pathway between 2.75 mM and 25 mM glucose. This was reflected by the increased ratio of phosphorylated AKT to total AKT (pAKT/AKT) (*p* < 0.001), phosphorylated mTOR to total mTOR (pmTOR/mTOR) (*p* < 0.0001), phosphorylated P70S6K to total P70S6K (pP70S6K/P70S6K) (*p* < 0.0001) and phosphorylated S6 to total S6 (pS6/S6) (*p* < 0.0001) (Figure 2). At 25 mM glucose, resveratrol led to increased ratios of pAKT/AKT and pP70S6K/P70S6K (*p* < 0.0001) (Figure 2A,C). At 5.5 mM glucose, resveratrol led to increased ratios of pP70S6K/P70S6K (*p* < 0.0001) and pS6/S6 (*p* = 0.016) (Figure 2C,D). Resveratrol did not affect the pmTOR/mTOR ratio at any glucose concentration (*p* > 0.05) (Figure 2B). Taken together, these results suggest that resveratrol does not activate the mTOR signaling pathway, except for P70S6K and S6.

### 3.3. Effect of Resveratrol on Circadian Rhythms in Myotubes

**Figure 3:**
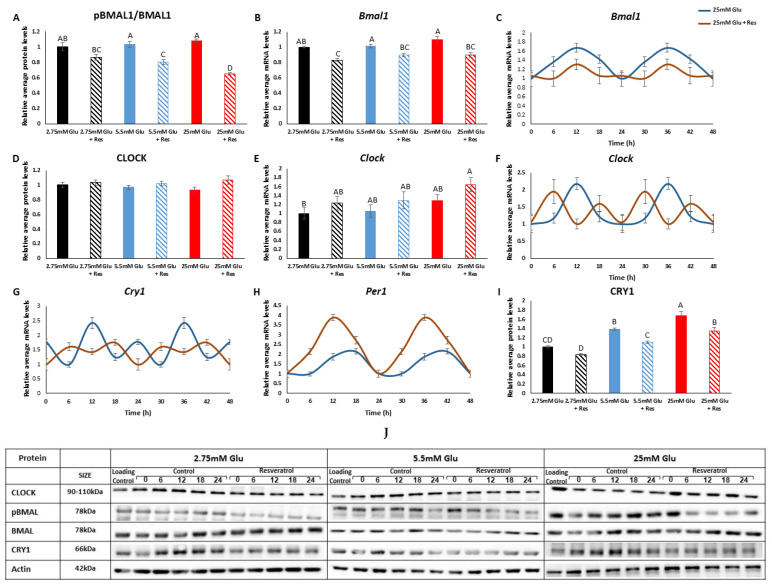
Effect of resveratrol on clock components. (**A**) pBMAL1/BMAL1. (**B**) *Bmal1* mRNA levels. (**C**) *Bmal1* mRNA oscillation. (**D**) CLOCK protein. (**E**) *Clock* mRNA levels. (**F**) *Clock* mRNA oscillation. (**G**) *Cry1* mRNA oscillation. (**H**) *Per1* mRNA oscillation. (**I**) CRY1. (**J**) Representative Western blots of all proteins at different glucose concentrations around the circadian cycle. C2C12 myotubes were incubated with resveratrol for 6 h and analyzed for an additional 24 h. Western blots and real-time quantitative PCR were performed to determine protein and mRNA levels, respectively. All time-points are expressed as average daily levels. Oscillations are presented as double plots. Within each panel, protein and gene levels display statistical significance (*p* < 0.05) only when bars are presented with different letters.

We next measured the levels of the clock transcription factor BMAL1, which turns into a translation factor once phosphorylated by the mTOR signaling pathway [19]. In agreement with the reduced mTOR activity, the ratio of phosphorylated BMAL1 to total BMAL1 (pBMAL1/BMAL1) also decreased (*p* < 0.001) in the presence of resveratrol (Figure 3A). *Bmal1* mRNA oscillated robustly in myotubes (*p* < 0.05, CircWave), but resveratrol led to a phase advance and a decrease in its amplitude and levels (Figure 3B,C). The levels of CLOCK protein and *Clock* mRNA did not change at the different glucose concentrations or resveratrol treatment (*p* > 0.05) (Figure 3D,E). Resveratrol treatment led to a phase advance in *Clock* mRNA (Figure 3F). Resveratrol also led to a phase advance of *Cry1* and *Per1* mRNA (Figure 3G,H). At increasing glucose concentrations, CRY1 protein levels increased (*p* < 0.0001), whereas resveratrol treatment led to a decrease (*p* < 0.0001) (Figure 3I). Taken together, these results suggest that resveratrol leads to advanced circadian rhythms and reduced levels of pBMAL1 and CRY1.

### 3.4. Effect of Resveratrol on Myotube Development

**Figure 4:**
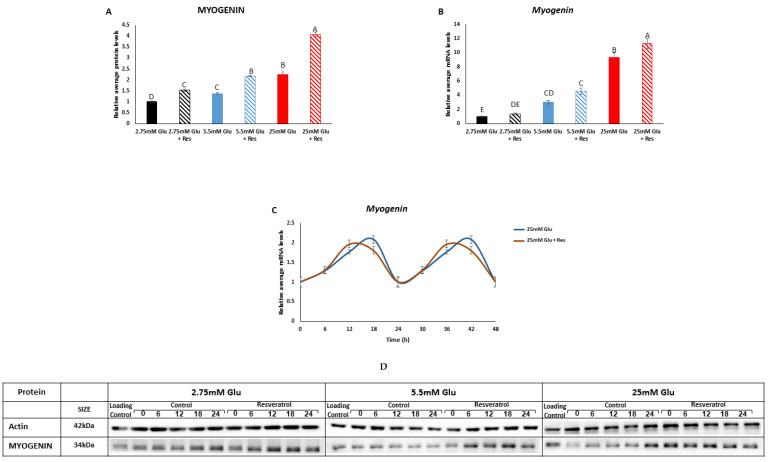
Effect of resveratrol on muscle development. (**A**) MYOGENIN levels. (**B**) *Myogenin* mRNA levels. (**C**) *Myogenin* mRNA oscillation. (**D**) Representative Western blots of all proteins at different glucose concentrations around the circadian cycle. C2C12 myotubes were incubated with resveratrol for 6 h and analyzed for an additional 24 h. Western blots and real-time quantitative PCR were performed to determine protein and mRNA levels, respectively. All time-points are expressed as average daily levels. Oscillations are presented as double plots. Within each panel, protein and gene levels display statistical significance (*p* < 0.05) only when bars are presented with different letters.

We next analyzed the effect of increasing concentrations of glucose in the presence or absence of resveratrol on myogenin, which is a marker of myotubes development. MYOGENIN protein and *Myogenin* mRNA levels increased proportionally to glucose concentrations (*p* < 0.0001) (Figure 4A,B). At each glucose concentration, resveratrol led to an additional increase in MYOGENIN (*p* < 0.0001) (Figure 4A,B). Resveratrol treatment led to a phase advance in *Myogenin* mRNA expression (Figure 4C). Taken together, these results suggest that resveratrol increases myogenin expression and advances its rhythms.

## 4. Discussion

In this study, we investigated the circadian metabolic effect of resveratrol on C2C12 myotubes under increasing concentrations of glucose. We show that myotubes treated with increasing concentrations of glucose have increased mTOR and reduced AMPK signaling. Resveratrol treatment in combination with increasing concentrations of glucose affected these signaling pathways, by activating the SIRT1-AMPK-PP2A axis and inhibiting the mTOR pathway. In addition, resveratrol led to a phase advance in circadian rhythms and increased myogenin expression.

Activation of the mTOR signaling pathway and inhibition of AMPK signaling under increasing concentrations of glucose is expected as high energy levels lead to the induction of anabolic pathways and suppressed catabolic pathways [20]. The reduced activation of AMPK under increasing glucose concentrations was mirrored by increased activation of its target ACC, the rate-limiting enzyme in fatty acid synthesis. ACC is expected to be activated under increasing glucose concentrations as it generates malonyl CoA for the synthesis of fatty acids under conditions of ample energy [21]. However, resveratrol modified the cellular metabolic state. Resveratrol has been reported to activate SIRT1 [10]. SIRT1 activation is usually accompanied by AMPK activation [13]. Indeed, studies report that resveratrol leads to AMPK activation by phosphorylation [15]. PP2A has been reported to dephosphorylate AMPK leading to inactivation of the latter [17,18]. However, herein, we show that resveratrol treatment resulted in reduced levels of phosphorylated Tyr307 on PP2A, the inhibitory site, thereby increasing the activity of PP2A. This effect of resveratrol on PP2A has also been documented in several recent publications [16,22,23]. However, these studies did not measure AMPK activation in parallel. In studies in which both AMPK and PP2A were measured, the results depended on the cell type. In hepatocytes, resveratrol treatment led to PP2A activation and AMPK inhibition [24], which emphasizes the reported role of PP2A in the inactivation of AMPK [25]. We recently also reported that resveratrol shifts hepatocytes to the fasting state by increasing the activity of PP2A, reducing AMPK activity and inducing the expression of the key gluconeogenic enzyme phosphoenolpyruvate carboxykinase (PEPCK) [8]. In contrast, but similarly to our findings herein, in a rat brain, it was found that both AMPK and PP2A were activated by resveratrol leading to Tau dephosphorylation [26]. In addition, numerous studies have shown that PP2A activity can be increased by AMPK-mediated phosphorylation [27,28]. Thus, in the brain and muscle cells, resveratrol leads to both activation of PP2A and AMPK, which emphasizes the fact that AMPK may not be the main target of PP2A, but rather vice versa, in these cell types.

Resveratrol did not affect mTOR at any glucose concentration and led to AKT activation only at very high glucose concentrations. These findings are consistent with previous publications that showed that, upon PP2A activation, AKT was inhibited [29,30]. Interestingly, resveratrol led to P70S6K and S6 activation even at physiological glucose concentrations. These findings are supported in the literature, as it has been shown that P70S6K and S6 can be activated in an mTOR-independent manner in C2C12 myotubes [31,32,33,34]. Activation of P70S6K has been shown to mediate muscle differentiation and hypertrophy [35]. Indeed, our results show that resveratrol increases myogenin expression compared to glucose treatment alone. Although a change in morphology could not be detected, presumably because the effect is for the long run, it was shown that myogenin is crucial for myotube differentiation and development [36]. More specifically, the absence of myogenin has been shown to lead to loss of skeletal muscles, despite the presence of normal committed myoblasts [37]. These findings imply that there is no compensation for myogenin absence, and it has an indispensable role in terminal differentiation of myoblasts [38].

Resveratrol treatment led to advanced circadian rhythms and reduced levels of pBMAL1 and CRY1. These changes are congruent with other reports that show that altered AMPK and mTOR activity leads to modified circadian rhythms [39,40]. It has been shown that activation of the mTOR pathway leads to a rhythmic phosphorylation of BMAL1, allowing it to associate with the translational machinery as well as stimulate circadian oscillations of protein synthesis [19]. Conversely, as a transcription factor and a core component of the circadian clock, BMAL1 has the capacity to function as a negative regulator of mTORC1 signaling [39]. The diminished mTOR signaling pathway, and consequently the reduced levels of phosphorylated BMAL1 observed in this study, align with previous reports, indicating that BMAL1 remains a transcriptional activator of the core clock mechanism, leading to phase advances rather than functioning as a translational factor. These findings are consistent with our earlier observations in hepatocytes [8]. In addition, SIRT1 activation has been shown to deacetylate histones and activate the CLOCK:BMAL1-mediated transcription [12]. In addition, the levels of CRY1, a member of the core clock negative feedback loop, were reduced, most probably as a result of the activation of AMPK. AMPK has been shown to phosphorylate and, as a result, lead to the degradation of CRY1 [41]. Thus, both the reduced levels of pBMAL1 and CRY1 may promote the activity of the positive loop of the clock, leading to advanced rhythms.

**Figure 5:**
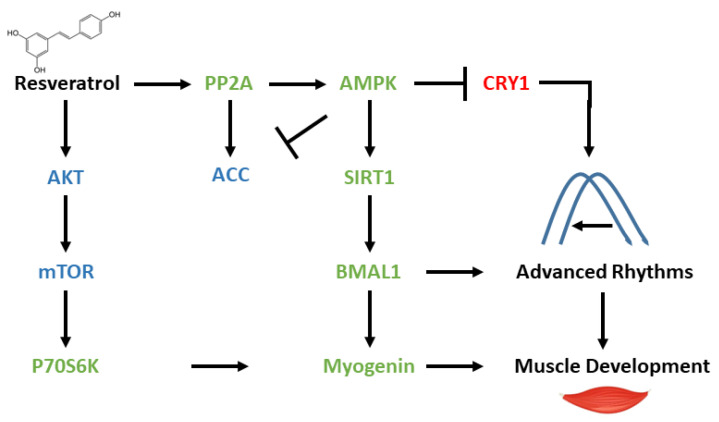
Metabolic and circadian effect of resveratrol in myotubes. Resveratrol leads to the activation of AMPK and PP2A. AMPK leads on the one hand to the degradation of CRY1 and on the other hand to the activation of SIRT1. SIRT1 histone deacetylation activates BMAL1, which induces *Myogenin* gene expression. Myogenin is also induced by P70S6K, which is activated in an mTOR-independent manner. MYOGENIN expression leads to muscle development. Low mTOR signaling leads to reduced pBMAL1, allowing BMAL1 to work as a transcription factor, rather than a translation factor. BMAL1 activation as well as CRY1 degradation leads to advanced circadian rhythms. Red—inhibited, green—activated, blue—unchanged.

Studies have shown that the expression of myogenin is under circadian control [42,43,44,45]. Myogenin expression peaks at specific times of the day, which align with periods of increased muscle repair and regeneration activity [42,45]. Our results suggest that resveratrol led to increased clock functionality and phase advances. As the clock controls myogenin expression and myogenin is required for muscle development, we surmise that resveratrol leads to muscle development via clock induction and, as a result, myogenin induced expression (Figure 5).

## 5. Conclusions

Herein we show that resveratrol leads to the activation of the SIRT1-AMPK-PP2A signaling pathway (Figure 5). AMPK activation decreases the activity of mTOR phosphorylation, which, in turn, leads to reduced pBMAL1, allowing BMAL1 to work as a transcription factor, rather than a translation factor. In addition, AMPK activation leads to CRY1 degradation, which relieves the inhibition from the CLOCK:BMAL1-mediated expression. BMAL1 activity, as a transcription factor, as well as reduced levels of negative feedback loop member CRY1, lead to phase advances in circadian expression (Figure 5). Activation of P70S6K, independent of the mTOR signaling pathway, leads to myogenin expression and muscle development. The mechanism by which resveratrol affects metabolic and circadian rhythms in different cell types merits further investigation.

## Data Availability

The datasets generated during and/or analyzed during the current study are available from the corresponding author on reasonable request.

## Supplementary Materials

The following supporting information can be downloaded at: https://www.mdpi.com/article/10.3390/cells13121069/s1, Table S1: RT primers.

## References

[1] Devlin P.F. (2002). Signs of the time: Environmental input to the circadian clock. J. Exp. Bot..

[2] Albrecht U. (2012). Timing to perfection: The biology of central and peripheral circadian clocks. Neuron.

[3] Eckel-Mahan K., Sassone-Corsi P. (2013). Metabolism and the circadian clock converge. Physiol. Rev..

[4] Froy O. (2010). Metabolism and circadian rhythms—Implications for obesity. Endocr. Rev..

[5] Froy O., Garaulet M. (2018). The Circadian Clock in White and Brown Adipose Tissue: Mechanistic, Endocrine, and Clinical Aspects. Endocr. Rev..

[6] Ramírez-Garza S.L., Laveriano-Santos E.P., Marhuenda-Muñoz M., Storniolo C.E., Tresserra-Rimbau A., Vallverdú-Queralt A., Lamuela-Raventós R.M. (2018). Health Effects of Resveratrol: Results from Human Intervention Trials. Nutrients.

[7] Mikstacka R., Rimando A.M., Ignatowicz E. (2010). Antioxidant effect of trans-resveratrol, pterostilbene, quercetin and their combinations in human erythrocytes in vitro. Plant Foods Hum. Nutr..

[8] Chatam O., Chapnik N., Froy O. (2022). Resveratrol Induces the Fasting State and Alters Circadian Metabolism in Hepatocytes. Plant Foods Hum. Nutr..

[9] Spaleniak W., Cuendet M. (2023). Resveratrol as a circadian clock modulator: Mechanisms of action and therapeutic applications. Mol. Biol. Rep..

[10] Tsai H.Y., Ho C.T., Chen Y.K. (2017). Biological actions and molecular effects of resveratrol, pterostilbene, and 3′-hydroxypterostilbene. J. Food Drug Anal..

[11] Okada Y., Okada M. (2020). Quercetin, caffeic acid and resveratrol regulate circadian clock genes and aging-related genes in young and old human lung fibroblast cells. Mol. Biol. Rep..

[12] Chang H.C., Guarente L. (2013). SIRT1 mediates central circadian control in the SCN by a mechanism that decays with aging. Cell.

[13] Sharma A., Anand S.K., Singh N., Dwivedi U.N., Kakkar P. (2023). AMP-activated protein kinase: An energy sensor and survival mechanism in the reinstatement of metabolic homeostasis. Exp. Cell Res..

[14] Martinet W., De Loof H., De Meyer G.R.Y. (2014). mTOR inhibition: A promising strategy for stabilization of atherosclerotic plaques. Atherosclerosis.

[15] Lan F., Weikel K.A., Cacicedo J.M., Ido Y. (2017). Resveratrol-Induced AMP-Activated Protein Kinase Activation Is Cell-Type Dependent: Lessons from Basic Research for Clinical Application. Nutrients.

[16] Hecht J.T., Coustry F., Veerisetty A.C., Hossain M.G., Posey K.L. (2021). Resveratrol Reduces COMPopathy in Mice Through Activation of Autophagy. JBMR Plus.

[17] Guo S., Chen C., Ji F., Mao L., Xie Y. (2017). PP2A catalytic subunit silence by microRNA-429 activates AMPK and protects osteoblastic cells from dexamethasone. Biochem. Biophys. Res. Commun..

[18] Perera N.D., Sheean R.K., Scott J.W., Kemp B.E., Horne M.K., Turner B.J. (2014). Mutant TDP-43 deregulates AMPK activation by PP2A in ALS models. PLoS ONE.

[19] Lipton J.O., Yuan E.D., Boyle L.M., Ebrahimi-Fakhari D., Kwiatkowski E., Nathan A., Güttler T., Davis F., Asara J.M., Sahin M. (2015). The Circadian Protein BMAL1 Regulates Translation in Response to S6K1-Mediated Phosphorylation. Cell.

[20] Keerthana C.K., Rayginia T.P., Shifana S.C., Anto N.P., Kalimuthu K., Isakov N., Anto R.J. (2023). The role of AMPK in cancer metabolism and its impact on the immunomodulation of the tumor microenvironment. Front. Immunol..

[21] Luo D.X., Tong D.J., Rajput S., Wang C., Liao D.F., Cao D., Maser E. (2012). Targeting acetyl-CoA carboxylases: Small molecular inhibitors and their therapeutic potential. Recent. Pat. Anticancer Drug. Discov..

[22] Schweiger S., Matthes F., Posey K., Kickstein E., Weber S., Hettich M.M., Pfurtscheller S., Ehninger D., Schneider R., Krauß S. (2017). Resveratrol induces dephosphorylation of Tau by interfering with the MID1-PP2A complex. Sci. Rep..

[23] Liu C., Zhang R., Sun C., Zhang H., Xu C., Liu W., Gao W., Huang S., Chen L. (2015). Resveratrol prevents cadmium activation of Erk1/2 and JNK pathways from neuronal cell death via protein phosphatases 2A and 5. J. Neurochem..

[24] Lu C., Xing H., Yang L., Chen K., Shu L., Zhao X., Song G. (2021). Resveratrol Ameliorates High-Fat-Diet-Induced Abnormalities in Hepatic Glucose Metabolism in Mice via the AMP-Activated Protein Kinase Pathway. Evid. Based Complement. Alternat. Med..

[25] Joseph B.K., Liu H.Y., Francisco J., Pandya D., Donigan M., Gallo-Ebert C., Giordano C., Bata A., Nickels J.T. (2015). Inhibition of AMP Kinase by the Protein Phosphatase 2A Heterotrimer, PP2APpp2r2d. J. Biol. Chem..

[26] Shati A.A., Alfaifi M.Y. (2019). Trans-resveratrol Inhibits Tau Phosphorylation in the Brains of Control and Cadmium Chloride-Treated Rats by Activating PP2A and PI3K/Akt Induced-Inhibition of GSK3beta. Neurochem. Res..

[27] Kim K.Y., Baek A., Hwang J.E., Choi Y.A., Jeong J., Lee M.S., Cho D.H., Lim J.S., Kim K.I., Yang Y. (2009). Adiponectin-activated AMPK stimulates dephosphorylation of AKT through protein phosphatase 2A activation. Cancer Res..

[28] Chen B., Li J., Zhu H. (2016). AMP-activated protein kinase attenuates oxLDL uptake in macrophages through PP2A/NF-kappaB/LOX-1 pathway. Vascul. Pharmacol..

[29] Hong K., Lou L., Gupta S., Ribeiro-Neto F., Altschuler D.L. (2008). A novel Epac-Rap-PP2A signaling module controls cAMP-dependent Akt regulation. J. Biol. Chem..

[30] Tohmé R., Izadmehr S., Gandhe S., Tabaro G., Vallabhaneni S., Thomas A., Vasireddi N., Dhawan N.S., Ma’ayan A., Sharma N. (2019). Direct activation of PP2A for the treatment of tyrosine kinase inhibitor-resistant lung adenocarcinoma. JCI Insight.

[31] Duan Y., Li F., Li Y., Tang Y., Kong X., Feng Z., Anthony T.G., Watford M., Hou Y., Wu G. (2016). The role of leucine and its metabolites in protein and energy metabolism. Amino Acids.

[32] Alway S.E., Pereira S.L., Edens N.K., Hao Y., Bennett B.T. (2013). beta-Hydroxy-beta-methylbutyrate (HMB) enhances the proliferation of satellite cells in fast muscles of aged rats during recovery from disuse atrophy. Exp. Gerontol..

[33] Pimentel G.D., Rosa J.C., Lira F.S., Zanchi N.E., Ropelle E.R., Oyama L.M., Oller do Nascimento C.M., de Mello M.T., Tufik S., Santos R.V. (2011). beta-Hydroxy-beta-methylbutyrate (HMbeta) supplementation stimulates skeletal muscle hypertrophy in rats via the mTOR pathway. Nutr. Metab..

[34] Eley H.L., Russell S.T., Baxter J.H., Mukerji P., Tisdale M.J. (2007). Signaling pathways initiated by beta-hydroxy-beta-methylbutyrate to attenuate the depression of protein synthesis in skeletal muscle in response to cachectic stimuli. Am. J. Physiol. Endocrinol. Metab..

[35] Sakushima K., Yoshikawa M., Osaki T., Miyamoto N., Hashimoto T. (2020). Moderate hypoxia promotes skeletal muscle cell growth and hypertrophy in C2C12 cells. Biochem. Biophys. Res. Commun..

[36] Asfour H.A., Allouh M.Z., Said R.S. (2018). Myogenic regulatory factors: The orchestrators of myogenesis after 30 years of discovery. Exp. Biol. Med..

[37] Hasty P., Bradley A., Morris J.H., Edmondson D.G., Venuti J.M., Olson E.N., Klein W.H. (1993). Muscle deficiency and neonatal death in mice with a targeted mutation in the myogenin gene. Nature.

[38] Myer A., Olson E.N., Klein W.H. (2001). MyoD cannot compensate for the absence of myogenin during skeletal muscle differentiation in murine embryonic stem cells. Dev. Biol..

[39] Dadon-Freiberg M., Chapnik N., Froy O. (2020). REV-ERBalpha activates the mTOR signalling pathway and promotes myotubes differentiation. Biol. Cell.

[40] Lee Y., Kim E.K. (2013). AMP-activated protein kinase as a key molecular link between metabolism and clockwork. Exp. Mol. Med..

[41] Lamia K.A., Sachdeva U.M., DiTacchio L., Williams E.C., Alvarez J.G., Egan D.F., Vasquez D.S., Juguilon H., Panda S., Shaw R.J. (2009). AMPK regulates the circadian clock by cryptochrome phosphorylation and degradation. Science.

[42] Andrews J.L., Zhang X., McCarthy J.J., McDearmon E.L., Hornberger T.A., Russell B., Campbell K.S., Arbogast S., Reid M.B., Walker J.R. (2010). CLOCK and BMAL1 regulate MyoD and are necessary for maintenance of skeletal muscle phenotype and function. Proc. Natl. Acad. Sci. USA.

[43] Bozek K., Relógio A., Kielbasa S.M., Heine M., Dame C., Kramer A., Herzel H. (2009). Regulation of clock-controlled genes in mammals. PLoS ONE.

[44] Harfmann B.D., Schroder E.A., Esser K.A. (2015). Circadian rhythms, the molecular clock, and skeletal muscle. J. Biol. Rhythms.

[45] Shavlakadze T., Anwari T., Soffe Z., Cozens G., Mark P.J., Gondro C., Grounds M.D. (2013). Impact of fasting on the rhythmic expression of myogenic and metabolic factors in skeletal muscle of adult mice. Am. J. Physiol. Cell Physiol..

