# Supplementary material for: Resveratrol Induces Myotube Development by Altering Circadian Metabolism via the SIRT1-AMPK-PP2A Axis
Source: Cells. 2024 Jun 20;13(12):1069. doi: 10.3390/cells13121069 (PMC11201382; doi:10.3390/cells13121069)
Supplement: Supplementary file 1 [file cells-13-01069-s001.zip › cells-3027458-supplementary.pdf]

Supplementary Table S1: Quantitative real-time PCR primer sequences

Gene	Forward primer	Reverse primer
Actin	5'-CTAAGGCCAACCGTGAAAAG-3'	5'-GGGGTGTTGAAGGTCTCAAA-3'
Bmal1	5'-CAAGAATGCAAGGGAGGCC-3'	5'-TTGTCCCGACGCCTCTTTT-3'
Clock	5'-CCTAGAAAATCTGGCAAAATGTCA-3'	5'-CCTTTCCATATTGCATTAAGTGCT-3'
Cry1	5'-AGGCAGCTGATGTATTTCCCA-3'	5'-AGTTTAGTGATGTTCCATTCCTTGAA-3'
Myogenin	5'-GGCAATGCACTGGAGTTCG-3'	5'-AGCCGCGAGCAAATGATC-3'
Per1	5'-CCGAATACACACTTCGAACCAG-3'	5'-TCCCGTTTGCAACGCAG-3'
